# Comparing ensemble methods combined with different aggregating models using micrograph cell segmentation as an initial application example

**DOI:** 10.1016/j.jpi.2023.100304

**Published:** 2023-03-05

**Authors:** St. Göb, S. Sawant, F.X. Erick, C. Schmidkonz, A. Ramming, E.W. Lang, T. Wittenberg, Th.I. Götz

**Affiliations:** a Fraunhofer Institute for Integrated Circuits IIS, Erlangen, Germany; b Chair of Visual Computing, Friedrich-Alexander-Universität Erlangen-Nürnberg, Erlangen, Germany; c Department of Nuclear Medicine, Friedrich-Alexander-Universität Erlangen-Nürnberg and University Hospital Erlangen, Erlangen, Germany; d Department of Internal Medicine, University Hospital Erlangen, Erlangen, Germany; e CIML Group, Biophysics, University of Regensburg, Regensburg, Germany; f Department of Industrial Engineering and Health, Technical University of Applied Sciences Amberg-Weiden, Weiden, Germany

**Keywords:** Deep neural networks, Alpha-stable function, Combine ensembles, Cell segmentation

## Abstract

Strategies such as *ensemble learning* and *averaging techniques* try to reduce the variance of single deep neural networks. The focus of this study is on *ensemble averaging* techniques, fusing the results of differently initialized and trained networks. Thereby, using micrograph cell segmentation as an application example, various ensembles have been initialized and formed during network training, whereby the following methods have been applied: (a) random seeds, (b) *L*_1_-norm pruning, (c) variable numbers of training examples, and (d) a combination of the latter 2 items. Furthermore, different averaging methods are in common use and were evaluated in this study. As averaging methods, the *mean*, the *median,* and the *location parameter* of an *alpha-stable distribution*, fit to the histograms of class membership probabilities (CMPs), as well as a *majority* vote of the members of an ensemble were considered. The performance of these methods is demonstrated and evaluated on a micrograph cell segmentation use case, employing a common state-of-the art deep convolutional neural network (DCNN) architecture exploiting the principle of the common VGG-architecture. The study demonstrates that for this data set, the choice of the ensemble averaging method only has a marginal influence on the evaluation metrics (accuracy and Dice coefficient) used to measure the segmentation performance. Nevertheless, for practical applications, a simple and fast estimate of the mean of the distribution is highly competitive with respect to the most sophisticated representation of the CMP distributions by an alpha-stable distribution, and hence seems the most proper ensemble averaging method to be used for this application.

## Introduction

Ensemble learning attempts to improve the performance of single deep neural network models by *averaging techniques* for different application use cases such as detection, classification, or segmentation of objects. Ensemble averaging tries to solve the so-called bias–variance dilemma of neural networks^1^ by creating a deep neural network (DNN) *ensemble* with a large number of diverse DNN realizations to reduce bias and then forming a possibly weighted average to reduce the variance. Additionally, for such image-based applications, ensemble learning provides effective means to improve the accuracy and robustness of DNN predictions of pixelwise class membership probabilities (CMPs). In literature, different strategies are reported to combine predictions from an ensemble of component networks. Starting with simpler methods like calculating the mean or extremes (maximum, minimum)^2^^,^^3^ of CMPs, more complex ensembles of weighted network predictions use stochastic search methods such as simulated annealing.^4^ Nonetheless the averaging method^5^^,^^6^ and majority voting are the ones used most often.^7^^,^^8^ Usually, results only improve with a larger number of ensemble members.

This study aims to compare methods of combining different DNN predictions from an ensemble. As an application use case, this study deploys the segmentation of biomedical micrograph cell images. Besides computing, the mean and median of the output distributions, majority voting is also employed. Furthermore, as the distributions of predicted CMPs often show skewed distributions, a parameterized Lévy *α*-stable distribution is fit to the corresponding histogram to represent the distributions analytically. Note that such distributions belong to the class of stable probability distributions, which are characterized by 4 parameters, a stability parameter 0 ≤ *α* ≤ 2, another shape parameter *β*, and a location and scale parameter *μ* and *c*, respectively. Further note that Cauchy (*α* = 1) and Gauss distributions (*α* = 2, *β* = 0) are special cases of *α*-stable distributions. Having an analytic description of the underlying CMP distribution offers the possibility to also provide a measure of reliability of the extracted statistic.

## Method

**Fig. 1:**
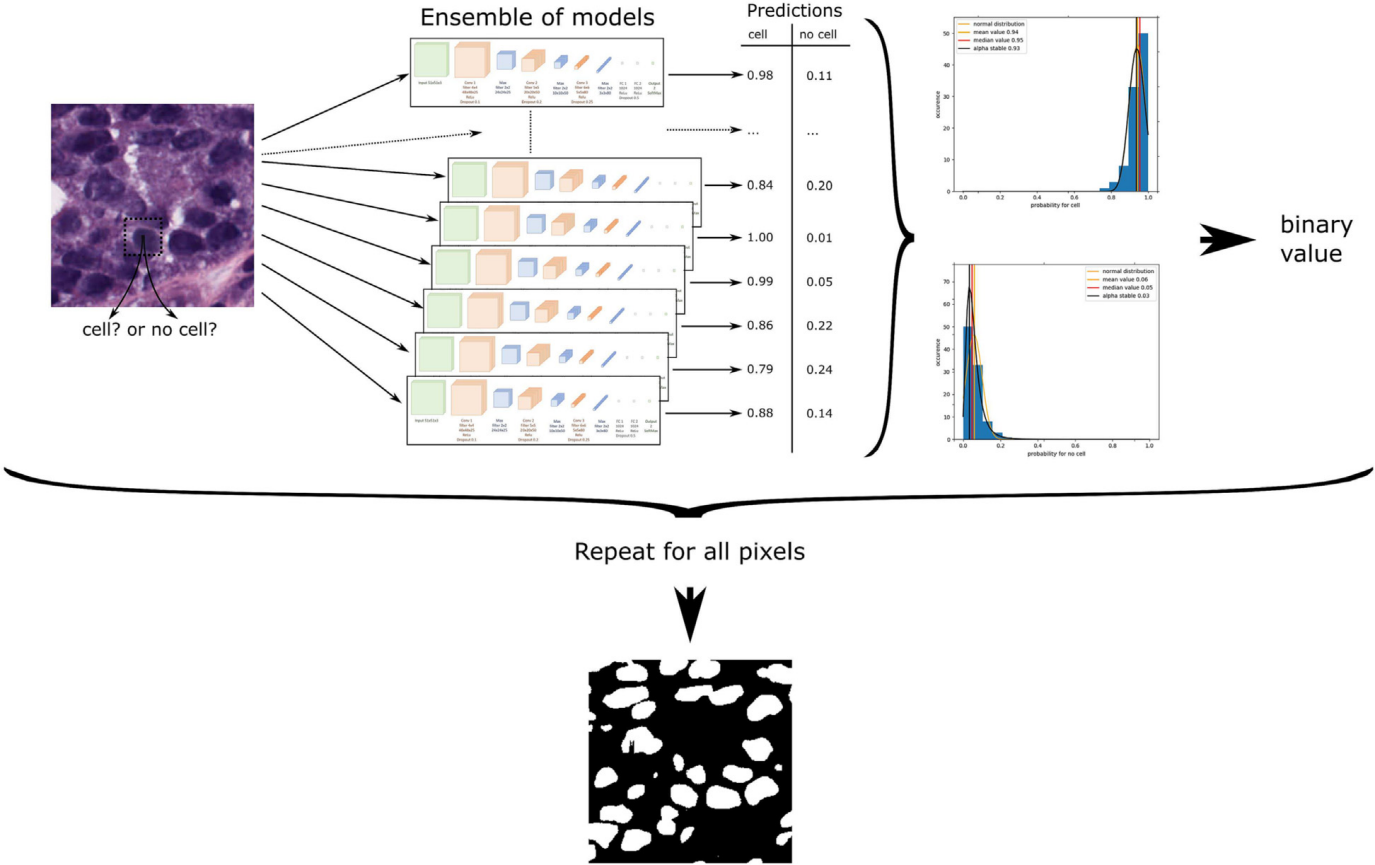
Technique to combine an ensemble of deep neural networks with an application of cell segmentation in micrograph images.

In most research on ensemble learning, ensembles are created from a very small number of models, e.g. *N*_models_ = 10_._^9^ However, it is generally known that the robustness and meaningfulness of a data distribution increases with the number of its members. Our first preliminary tests have shown that, especially when using a small amount of training data *N*_training_ < 1 000, the maximal success of ensembles is only achieved with a model number of more than *N*_models_ ∈ [60, …, 70] models. Any further increase in the number of participants does not have any negative effects onto the quality metrics. First of all, in this study, we analyzed how many neural networks we have to combine into an ensemble in order to get a statistically reliable result. For this reason in this study, ensembles of *N*_models_ = 100 deep neural network predictors are trained and then their predictions are combined in several ways to result in a related ensemble prediction. Fig. 1 provides an overview of the methodology. In general, for an ensemble of DNNs, some kind of data D (2D-images, image volumes, sound clips, text, feature vectors) are used as input. Then, the aim of every network *N*_*i*_, *i* ∈ [0, …, *N*_models_] is to classify the input with respect to a certain binary target or loss function *yes* (1) or *no* (0). Thus, an ensemble of *N*_models_ = 100 DNNs results in 100 individual CMPs, each one providing a probability how the input data D relates to the expected output. If these binary decisions are considered, the resulting probability distribution suggest different methods to binarize the CMPs. This binary result can then be compared with a known ground truth using some metrics, such as accuracy, Dice coefficient, Jaccard index, or others.

For our experiments, we use micrograph cell segmentation as an application example. Hence, small 51 × 51 pixel image patches of cell images serve as input to our DNNs. The aim of every network is to classify the center pixel of the patch as being either of class foreground (*cell*) or class background (*no cell*), respectively *yes* or *no* for the general case. An ensemble of *N*_models_ = 100 DNNs therefore result in 100 CMPs that the considered pixel represented either a foreground (*cell*) or background (*no cell*). An overview of all variables used in this publication is given in Table 6.

### Example data set

To evaluate our proposed approach—fusing the results of ensembles—the publicly available micrograph image data set of Kumar et al^10^ is used. This data collection consists of 30 cell micrograph images for training and 14 cell micrograph images for testing with a spatial resolution of 1 000 × 1 000 pixels and 3 color channels. The images have originally been captured with micoscopes of different vendors, while the slides with cell specimens have been collected from various clinics. All micrographs depict Hematoxylin- and Eosin-stained (*H* & *E*) tissues with cell assemblies from various organs (such as liver, colon, ...). Furthermore, for all cells respectively their cell nuclei, manually established ground-truth annotation is available for evaluation purposes. For our experiments, we only use a minor part of the data set of 2 images for pretraining, 7 images for training and validation, and 7 images for testing. For more information about the used images, the clinics, the diseases as well as the organs, refer to Appendix A. Examples of the micrograph images can be found in Kumar et al.^10^

The original task of the work by Kumar et al^10^ was ideally related to the automatic detection and delineation of all depicted cell-nuclei in micrographs, independent from the variations in staining, use of scanning systems and organs. As classical image segmentation methods such as thresholding, k-means, watersheds, region growing, are not suitable to solve this task generically, Kumar et al. have proposed a deep-neural network (DNN) approach. Within their approach, a deep convolutional neural network (DCNN) has been trained, which decides for a pixel (surrounded by the small image patch) if this pixel belongs to a certain foreground (*cell*) or the background (*no cell*). The selcted images from the data collection (see Appendix 8) are subdivided into overlapping image patches with size 51 × 51 × 3 pixels in 3 color channels yielding a total of 14 409 616 sub-images.

In our experiments, we used pretrained neural networks as well as networks trained from scratch and initialized with a random uniform distribution. To properly condition all model parameters for the training process, all neural networks have been *pretrained* employing image patches drawn from only 2 breast tissue micrographs cell images (for training and validation). Both images are obtained from the same scanner and were provided from the same hospital. Further training of the pretrained neural networks, as well as the training of the randomly initialized neural networks of the NN ensembles was pursued with 7 additional micrograph pairs (1 000 × 1 000 pixels) collected from different clinics and belonging to 1 out of 7 different tissue classes: breast, kidney, liver, prostate, bladder, colon, or stomach. For evaluation purposes, the training of the network ensembles was performed with data sets having different sizes. More specifically, the data sets encompassed *N*_1,training_ = 916, *N*_2,training_ = 9 159, and *N*_3,training_ = 91 593 image patches that were selected at random from the 7 cell images. Care was taken to ensure that each tissue class occurred equally frequently in every training data set, and all component networks of every ensemble employed the same training data set. The test data sets also consisted of 7 different cell images, each from one of the same tissue classes as the training data. In order to quantify the classification performance of the networks, all test data was predicted in each case. More detailed information on which images of the data set by Kumar et al^10^ were used can be found in the appendix in Table 7.

### Neural network architecture

The component networks of every ensemble were based on a deep convolutional neural network (DCNN) architecture in analogy with the work of Xing et al.^11^ This deep neural network has been designed for cell segmentation by classifying each pixel as foreground (“*cell*ˮ) or background (“*no cell*ˮ). In Table 1, the information about the network architecture is summarized. Overall, the network has 10 layers and containes *N*_p_ = 915 934 trainable parameters. The output of this deep neural network is a vector with 2 components representing the CMPs for the 2 classes, foreground (“*cell*ˮ) or background (“*no cell*ˮ). Note that from each presented 51 × 51 × 3 patch, only the center pixel is classified. As the sum of both CMPs needs to sum up to 1, the *binary cross-entropy loss function* was used for training the DCNN.

**Table 1:** Network architecture based on Xing et al.^11^

	Layer	Size	Filter size	Dropout	Activation
1	Input	51 × 51 × 3			
2	Convolution	48 × 48 × 25	4 × 4	0.1	ReLU
3	MaxPooling	24 × 24 × 25	2 × 2		
4	Convolution	20 × 20 × 50	5 × 5	0.2	ReLU
5	MaxPooling	10 × 10 × 50	2 × 2		
6	convolution	5 × 5 × 80	6 × 6	0.25	ReLU
7	MaxPooling	3 × 3 × 80	2 × 2		
8	FullyConnected	1 024		0.5	ReLU
9	FullyConnected	1 024		0.5	ReLU
10	Output	2			SoftMax

### *L*_1_ Filter pruning

To modify the model architecture, we use *L*_1_ filter pruning. The goal of filter pruning is to find out the importance of the various filters in the convolutional layer and systematically eliminating unimportant filters by deleting the corresponding weights. In detail, Kumar et al.^12^ describe the weights of a layer as {*W*^(*i*)^ ∈ R^*M*_*i*_×*N*_*i*_×*K*×*K*^, 1 ≤ *i* ≤ *L*}, where *W*^(*i*)^ represent the tensor of weights in the *i*-th layer with the number of input channels *M*_*i*_ and output channels *N*_*i*_ as well as hight and width of the kernel *K*. Thereby, *L* represents the *i*-th convolutional layer. Hence, pruning of a convolutional filter means deleting all the output channels *N*_*i*_ of a choosen input channel *M*_*i*_ and furthermore the input channels of the next layer *M*_*i*+1_. Mittal et al^13^ provide a good overview of filter pruning methods for this purpose. One of them is *L*_1_-filter pruning, where the *L*_1_-norm of each filter of a convolutional layer is computed and this single value gives an indication of its individual importance. The underlying assumption here is that these unimportant filters have no or only a small effect on the output of the network, and thus sorting these importance values by size provides a way to systematically prune filters. In this study, we use *L*_1_-pruning because it is fast to calculate, it gives good results compared to other pruning techniques, and does not need any other calculation than the *l*_1_-norm of every filter.

### DCNN ensemble learning

As mentioned before, both pretrained as well as randomly initialized networks were explored. For all training processes in this study, we applied a 70:30 split of the image patches for training and validation during the training process (according to Choo et al^14^). The pretrained DNNs were trained for 3 epochs, with cell images depicting breast tissue. Further training was performed by employing the remaining training image patches. The following ensemble learning methods have been considered in this study:

#### Random seeds

First, the *N*_*p*_ model parameters of all DCNN ensembles were initialized with random numbers with a uniform deviation distribution to start the training process. All networks of an ensemble were trained with an identical set of *N*_i,training_, *i* ∈ {1,2,3} training examples. This created an ensemble, where each component network has been initialized with a different seed.

#### *L*_1_ pruning

Second, to each of the 100 pretrained DCNNs with same seed, forming the ensemble, *L*_1_ filter pruning (See Section “L_1_ Filter pruningˮ) was applied. This *L*_1_ norm pruning is applied layer by layer to all 3 convolutional layers of the architecture, see section 2.3. The amount of pruning systematically varied from 0% to 50% in steps of 0.5%. This creates a DCNN ensemble, where each component network had been pruned differently.

#### Variable *N*_training_

As a third method of forming an ensemble, each of the 100 pretrained DCNNs with same seed was trained with a different amount *N*_training_ of training examples. The sizes for the training data sets were selected according to the following rule:

$$N_{4,\text{training}}=\left\{\begin{array}{ll} n\in \left\{1,\ldots ,19\right\} & 1\;000+\left(n-1\right)\cdot 500\\ n\in \left\{20,\ldots ,34\right\} & 10\;000+\left(n-19\right)\cdot 1\;000\\ n\in \left\{35,\ldots ,49\right\} & 25\;000+\left(n-34\right)\cdot 2\;000\\ n\in \left\{50,\ldots ,78\right\} & 55\;000+\left(n-49\right)\cdot 5\;000\\ n\in \left\{79,\ldots ,100\right\} & 200\;000+\left(n-78\right)\cdot 10\;000 \end{array}\right)$$

Here, *N*_4,training_ denotes the number of training examples, and *n* enumerates the component DNNs in the ensemble. In the following, the ensemble is called *E*(*N*_tr_). Detailed information about the explicit values of *N*_4,training_ is summarized in the Appendix in Table 8.

#### Variable *N*_training_-*L*_1_ pruning

As a variation to the previously mentioned ensembles *E*(*N*_tr_) with differently scaled training data, the idea was combined with an *L*_1_ pruning of the DCNN filter kernels and an amount of 80%. Please, see informations about pruning given in Section “L_1_ Filter pruningˮ.

Based on the ensemble learning strategies listed above, all in all, the following sets of different ensembles, each consisting of 100 trained networks, were computed. Remember that the total number of trainable parameters *N*_p_ in the original network architecture amounted to *N*_p_ = 915 934, thus affording at least a number of *N*_p_ = *N*_training_, matching each parameter at least with 1 training sample. At first, 3 ensembles were formed based on increasing numbers of observations *N*_training_ used for training and validation. Training of each component network in these ensembles was initialized with different seeds.

#### E(0.1%N_p_)

Each of the 100 networks in this ensemble was trained with an identical set of *N*_training_ = *N*_1,training_ = 10^−3^*N*_*p*_ = 916 observations. Hence, the number of training examples amounted to only 1 of the total number of model parameters of the DCNN.

#### E(1%N_p_)

Each network in this ensemble was trained with *N*_training_ = *N*_2,training_ = 10^−2^*N*_*p*_ = 9 159 observations, which corresponds to 1% of the number of adjustable model parameters.

#### E(10%N_p_)

Each network in this ensemble was trained with *N*_training_ = *N*_3,training_ = 10^−1^*N*_*p*_ = 91 593 observations, amounting to 10 % of the number of model parameters to be learned.

Next, 3 ensembles were formed where the filter kernels of all component DCNNs were *L*_1_-pruned during the training process. Thus to each of the 100 component DCNNs in this ensemble, *L*_1_ filter pruning was applied. During pruning systematically between 0.5% and 50% of the filter kernels were removed. All DCNNs were initialized with the same random seed.

#### E(0.1%N_p,_pru)

Based on the pretrained weights, *L*_1_-pruning was applied systematically to all filter kernels of the different convolutional layers. Hereby, between 0.5% and 50% of the filter kernels with the smallest weights were removed. Then, all component networks were fine tuned with an identical set of *N*_training_ = *N*_1,training_ = 10^−3^*N*_*p*_ = 916 observations. Note that each component network experienced a different amount of filter pruning, but all were trained with the same training data set.

#### E(1%N_p_, pru)

*L*_1_-pruning was applied systematically to all filter kernels of the different convolutional layers. Again between 0.5% and 50% of the filter kernels with the smallest weights were removed. All component networks were first trained with an identical set of *N*_training_ = *N*_2,training_ = 10^−2^*N*_*p*_ = 9 159 observations.

#### E(10%N_p_, pru)

*L*_1_-pruning was applied systematically to all filter kernels of the different convolutional layers and between 0.5% and 50% of the filter kernels with the smallest weights were removed. All component networks were first trained with an identical set of *N*_training_ = *N*_3,training_ = 10^−1^*N*_*p*_ = 91 593 observations.

Finally, 2 ensembles were formed combining network pruning with different sizes of the training data set for each component network. Note the difference to the ensembles before, where all component networks were trained with an identical training set size, which only differed from ensemble to ensemble. For initialization, a pretrained network was used.

#### E(N_tr_)

Each component network in this ensemble was trained with a different number of training examples according to *N*_4,training_. Every network had the original architecture with *N*_*p*_ = 915 934 adjustable parameters.

#### E(N_tr_, 80% pru)

Here, 80% of the filter kernels of every convolutional layer of the DCNN were pruned. Each component network was then trained with a different number of training sample points according to *N*_4,training_.

**Fig. 2:**
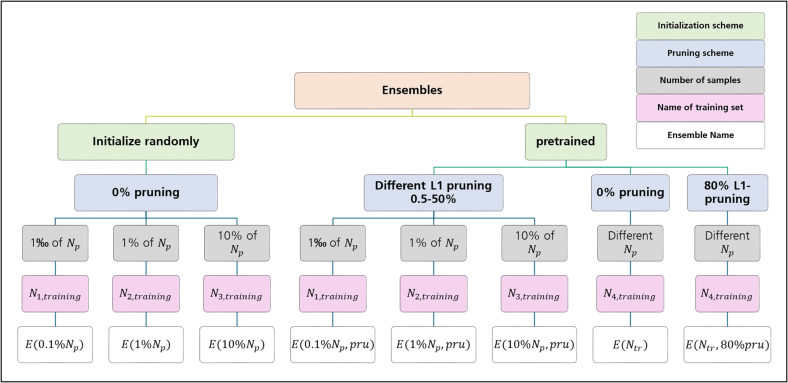
Overfiew of conducted experiments.

Fig. 2 gives a overview of the conducted experiments.

### Ensemble aggregating and binarization methods

For each ensemble, the training process delivered 100 CMPs *p*_*i*_, which are able to classify the central pixel of a yet unknown query patch *q* either as class foreground (“*cell*ˮ) or class background (“*no cell*ˮ). These resulting probability distributions for each network of each ensemble were tested for Gaussianity applying the Shapiro-Wilk test according to Shapiro and Wilk.^15^ Subsequently, 4 different ensemble averaging methods (*mean, median, majority vote, and alpha-stable fit*) were employed to convert the 100 CMPs of the individual DCNNs to the resulting prediction of the corresponding ensemble, i.e., into a binary classification map.

#### Mean value

First, the sample mean value over all probabilities was calculated, and then a threshold was used for binarization:

$$\mathrm{mean}\left(p_{i}\right)=\frac{1}{100}\sum_{i}^{100}p_{i}\left\{\begin{array}{c} >0.5\;\mathrm{set}\;\mathrm{to}\;1\\ \leq 0.5\;\mathrm{set}\;\mathrm{to}\;0 \end{array}\right)$$

#### Median

Second, the median over all probabilities was calculated, instead, to account for skewed distributions, and then a threshold was used for binarization:

$$\mathrm{median}\left(p_{i}\right)=\frac{1}{2}\left(\frac{1}{50}\sum_{i=1}^{50}p_{i}+\frac{1}{50}\sum_{i=50}^{100}p_{i}\right)\left\{\begin{array}{c} >0.5\;\mathrm{set}\;\mathrm{to}\;1\\ \leq 0.5\;\mathrm{set}\;\mathrm{to}\;0 \end{array}\right)$$

#### Majority vote

Third, a majority vote was calculated:

$$\mathrm{majority}\left(p_{i}\right)=\frac{n\left(p_{i}>0.5\right)}{n\left(p_{i}\leq 0.5\right)}\left\{\begin{array}{c} >1\;\mathrm{set}\;\mathrm{to}\;1\\ \leq 1\;\mathrm{set}\;\mathrm{to}\;0 \end{array}\right)$$

where *n*() denoted the number of CMPs *p*_*i*_, for which the condition inside the brackets was true.

#### Alpha-stable fit funtion

The histograms of CMPs were generally right skewed, thus being reminiscent of *α*-stable distributions (a class of Levy distributions).^16^, ^17^, ^18^, ^19^ Hence, we employed these functions to provide a smooth analytic description of the distribution of CMPs. The characteristic function *ϕ*(*ω*) of *α*-stable distributions is, in the frequency domain, given by

(1) $$\phi \left(\omega \right)=\phi_{0}^{\left(\alpha \right)}\left(\omega \right)\exp\left(\mathrm{i\mu \omega}\right)$$

where

(2) $$\phi_{0}^{\left(\alpha \right)}\left(\omega \right)=\left\{\begin{array}{cc} \exp\left(-{\left|\gamma \omega \right|}^{\alpha }\left[1-i\cdot \mathrm{sign}\left(\omega \right)\cdot \beta \cdot \tan\left(\alpha \frac{\pi }{2}\right)\right]\right), & \;\text{for}\;\left(\alpha \neq 1\right)\\ \exp\left(-|\gamma \omega |[1+i\cdot \mathrm{sign}\left(\omega \right)\cdot \frac{2}{\pi }\cdot \beta \cdot \log(|\omega |)]\right), & \;\text{for}\;\left(\alpha =1\right) \end{array}\right)$$

The *impulsiveness* parameter *α* ∈ (0, 2] provides a measure of tail thickness, while the *skewness β* ∈ [−1, +1] measures the degree and sign of asymmetry. Furthermore, *γ* > 0 denotes the *scale* parameter for dispersion, and the allocation parameter is set by *μ*. The latter can be seen analogously to the mean value of a Gaussian distribution, to which the alpha-stable distribution degenerates in case of *α* = 2, *β* = 0.

### Binary evaluation metrics

After training the ensembles of DNNs, the trained networks were deployed to segment the test data sample. From each trained DNN ensemble, we received a CMP value for each pixel in the test data. Then, post-processing methods convert these probabilities into binary values. The latter can then be compared to the known ground truth using appropriate evaluation metrics.

First, the average number of true positive TP (foreground (“*cell*ˮ) pixel correctly detected), true negative TN (background (“*no cell*ˮ) pixel correctly detected), false positive FP (a background (“*no cell*ˮ) pixel has wrongly been classified as foreground (“*cell*ˮ) pixel)) and false negative FN (a foreground (“*cell*ˮ) pixel has wrongly been classified as background (“*no cell*ˮ) pixel) pixels of the test data was calculated for each method of determining an ensemble vote from individual network predictions. The average prediction accuracy (ACC) is calculated from these values as follows

(3) $$\mathrm{ACC}=\frac{\mathrm{TP}+\mathrm{TN}}{\mathrm{TP}+\mathrm{FP}+\mathrm{TN}+\mathrm{FN}}$$

As second evaluation metric, we calculated the average Dice coefficient (DC), which is computed as

(4) $$\mathrm{DC}=\frac{2\mathrm{TP}}{2\mathrm{TP}+\mathrm{FP}+\mathrm{FN}}$$

Note the differences between the DC and the ACC. The latter also considers the true negatives, i.e., those pixels which were correctly predicted as belonging to class background (“*no cell*ˮ). The Dice coefficient rather weights more the number of true positives, i.e., the number of pixels that were correctly classified as belonging to class foreground (“*cell*ˮ). Note further that with cell images ∣*TP* ∣  ≪  ∣ *TN*∣ often holds, meaning the depicted cells are sparsely distributed in the images with respect to the background.

Because the predictions of the deep neural networks are continuous CMPs, we also computed both, a continuous Dice coefficient *cDC*^20^ and an accuracy measure *cACC*, according to

(5) $$\mathrm{cDC}=\frac{\sum_{i}a_{i}b_{i}}{\sum_{i}a_{i}}$$

(6) $$\mathrm{cACC}=\frac{\sum_{i}a_{i}b_{i}-\sum_{i}\left(1-a_{i}\right)\left(1-b_{i}\right)}{N}=\frac{2\sum_{i}a_{i}b_{i}-\sum_{i}a_{i}-\sum_{i}b_{i}-N}{N}$$

where the *a*_*i*_ denote elements of the ground truth, the *b*_*i*_ represent the combinations of predictions of the deep neural networks and *N* gives the total number of elements in the prediction. Eq. 5 is according to Shamir et al^20^ and Eq. 6 integrates the probabilities into the ACC formula analogously to the cDC.

## Results

### Evaluation metrics obtained from an individual DCNN

First, a single fully developed DCNN was trained, whereby 4 different sizes of the training data set have been employed (see Section “Methodˮ). Each time the training process converged, the trained DCNN was used to segment the entire set of test images. Based on these predictions, their ACC and DC were calculated. The evaluation metrics achieved high values and further increased with the number of training examples. The observed results are summarized in the Table 2.

**Table 2:** The metrics Accuracy (Acc), continous Accuracy (cAcc), Dice Coefficient (DC), and continuous Dice Coefficient (cDC) are given for an increasing amount of different numbers *N*_training_ of training examples.

*N*_training_	Acc	DC	cAcc	cDC
916	0.899	0.674	0.788	0.575
9 159	0.921	0.768	0.862	0.731
91 593	0.927	0.788	0.879	0.763
915 934	0.932	0.801	0.882	0.772

### Histograms of predicted CMP values

**Fig. 3:**
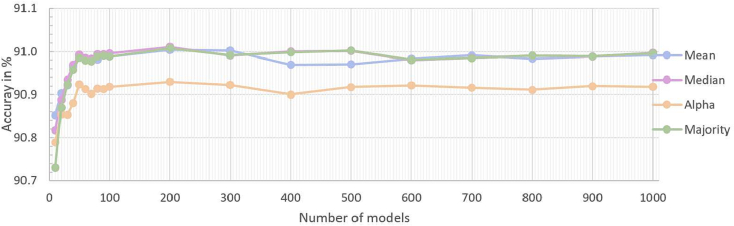
Test accuracy plotted against number of networks per ensemble for 916 training samples.

In order to determine a sufficient number of networks in an ensemble, between 10 and 1000 networks were combined into an ensemble and the accuracy for the test data was predicted. All networks were trained with 916 training data. A saturation curve of the accuracy is shown in Fig. 3. There were only minor changes in accuracy from 100 networks per ensemble. Therefore, we decided that further ensembles encompassing 100 neural networks.

In addition, all ensembles mentioned above, each encompassing 100 DCNNs, were trained and with them the test data was employed to predict corresponding CMPs of all pixels of the test images. For each pixel of every test image, 2 times 100 CMP values were obtained from every ensemble to assign a consensus CMP to every pixel of the test image.

**Fig. 4:**
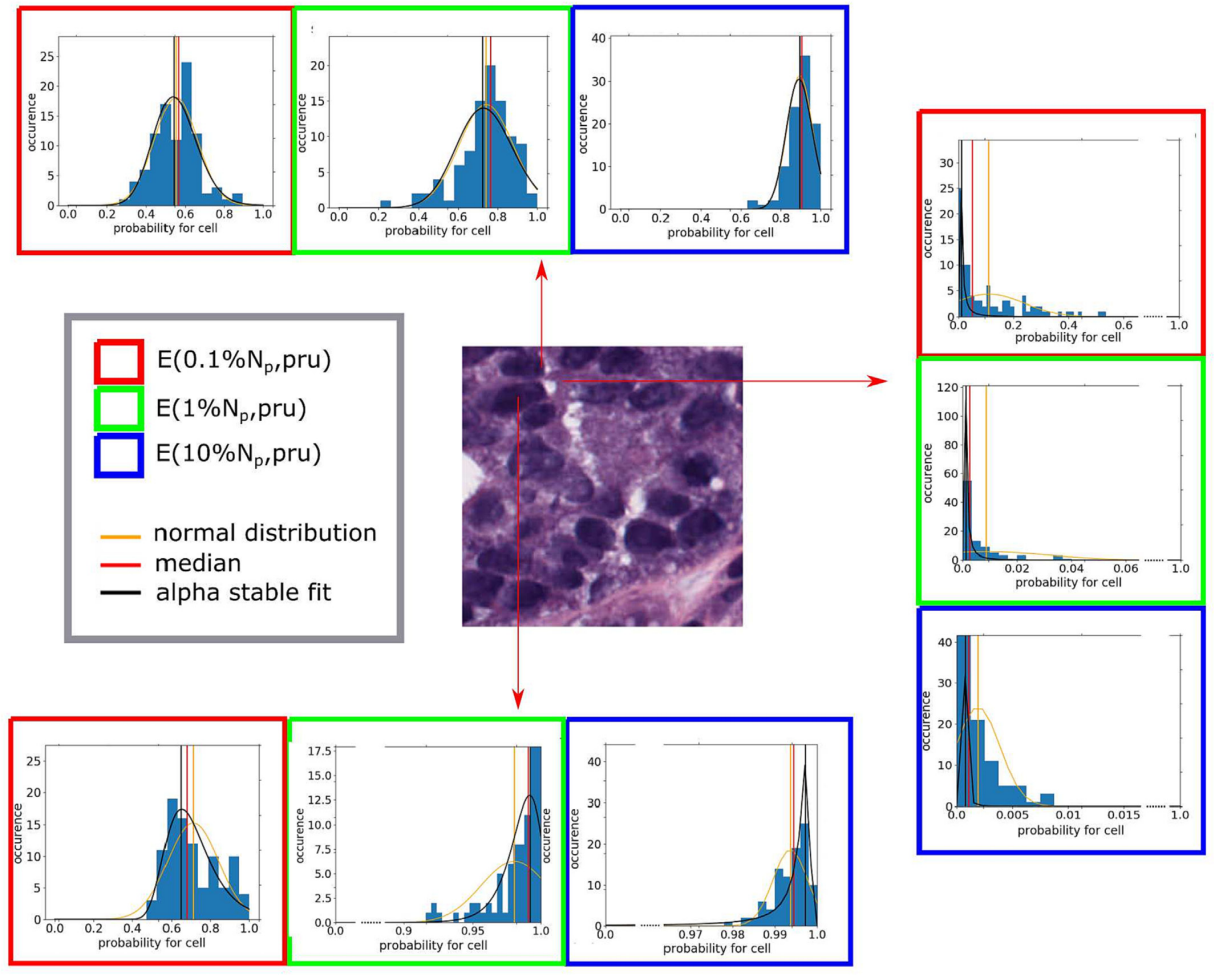
Examples of histograms of predicted CMP values from 100 ensembles, relating to class foreground (*cell*), of all image pixels, taken from three different *L*_1_-pruned ensembles.

The plotted histograms were fit to a Gaussian as well as an alpha-stable distribution. In Fig. 4, the histograms of CMP values are illustrated for 3 different ensembles. They all stem from the category *L*_1_-pruned, but also contain the effect of different training set sizes. The histograms became narrower with a higher number of training examples. This is a direct consequence of the improved precision of the network training. Only in case of the ensemble **E**(**1** % **N**_**p**_), the distribution resembles a Gaussian distribution according to a Shapiro-Wilk test. When comparing the computed statistics, namely mean value and location parameter, from both fitted distributions, it became apparent that they differed greatly from one another. Further note that only if the probability distribution is centered around a probability of 0.5, fluctuations have the chance to switch the related binary value from 0 → 1 and vice versa. Hence, only then the difference between the values can become discriminative.

### Average evaluation metrics obtained from ensembles

Histograms of CMPs were generated for each pixel of a test image and the metrics: mean value of a Gaussian distribution, median of the discrete set of CMPs, majority vote from all predicted CMPs, and location parameter of an alpha-stable distribution were then estimated. Based on their binarized equivalents, which were calculated using the various methods indicated above, average TP, TN, FP, and FN values were then computed with respect to class “foreground” (*cell*). The results are summarized in Table 3. It should be noted that these basic metrics are based on the same predicted CMPs. However, their quantitative values were determined by the method used to extract an ensemble prediction and resulted in different values of the binarized evaluation metrics.

**Table 3:** Quantitative basic classification statistics TP, TN, FP, and FN, averaged overall test images, and computed based on different ensemble learning and aggregating methods.

Ensemble	Mean value				Median			

TP	TN	FP	FN	TP	TN	FP	FN

*E*(0.1 % *N*_p_)	53 070	345 006	13 060	26 364	54 688	343 419	14 647	24 746
*E*(1 % *N*_p_)	64 352	340 931	17 135	15 082	65 002	340 030	18 036	14 432
*E*(10 % *N*_p_)	**65** **777**	340 942	17 124	13 657	**66** **038**	340 651	17 415	13 396
*E*(0.1 % *N*_p_, pru)	53 502	**344** **567**	13 499	25 932	55 126	**343** **027**	15 039	24 308
*E*(1 % *N*_p_, pru)	64 737	340 934	17 132	14 697	65 307	339 963	18 103	14 127
*E*(10 % *N*_p_, pru)	65 718	341 135	16 931	13 716	65 961	340 781	17 285	13 473
*E*(*N*_tr_)	65 421	342 518	15 548	14 013	66 012	341 807	16 259	13 422
*E*(*N*_tr_, 80 % pru)	65 371	342 453	15 613	14 063	65 937	341 805	16 261	13 497

Ensemble	Alpha				Majority vote			

TP	TN	FP	FN	TP	TN	FP	FN

*E*(0.1 % *N*_p_)	51 328	346 449	11 617	28 106	54 813	343 261	14 805	24 621
*E*(1 % *N*_p_)	63 186	342 245	15 821	16 248	65 121	339 891	18 175	14 313
*E*(10 % *N*_p_)	**65** **059**	341 723	16 343	14 375	**66** **107**	340 553	17 513	13 327
*E*(0.1 % *N*_p_, pru)	51 774	**346** **081**	11 985	27 660	55 126	**343** **027**	15 039	24 308
*E*(1 % *N*_p_, pru)	63 536	342 242	15 824	15 898	65 307	339 963	18 103	14 127
*E*(10 % *N*_p_, pru)	64 963	341 967	16 099	14 471	65 961	340 781	17 285	13 473
*E*(*N*_tr_)	63 896	343 992	14 074	15 538	66 111	341 669	16 397	13 323
*E*(*N*_tr_, 80 % pru)	63 611	344 017	14 049	15 823	66 029	341 700	16 366	13 405

All in all, only tiny differences in the metrics could be found between the ensemble averaging methods. This suggests that the probability distribution has large values only around a *p*_*i*_ = 0.5 in a few pixels. This is because only then, a difference between the methods could become visible in the binary evaluation metrics.

## Discussion

Let’s first have a look to the basic binary metrics TP, TN, FP, and FN. Note that positive values referred to class foreground (“*cell*ˮ) and negative values to class background (“*no cell*ˮ). Clearly, FP and FN should be as small as possible and TN was expected to be much larger than TP for microscopic cell images. At least the last relation was clearly reflected in the metrics collected in Table 3. The values of FP and FN were also much less than the number of TPs, Furthermore, *FP* = *FN* ≪ *TP* held across all ensembles except *E*(0.1 % *N*_p_) and *E*(0.1 % *N*_p_, pru). There *FN* ≈ 2*FP* held across all ensemble averaging methods. Considering the impact of ensemble learning methods on the metrics, it was found that the absolute values of the various metrics consistently increased with the number *N*_training_ of test observations in a non-linear fashion. The increase was stronger with small *N*_training_ but flattened considerably when *N*_training_ became large. These trends held true for the ensembles *E*(*x* % *N*_p_) and *E*(*x* % *N*_p_, pru) also across the different ensemble averaging methods. But the ensembles *E*(*N*_tr_) and *E*(*N*_tr_, 80 % pru) showed almost no effect of the rather strong network pruning on the quantitative metrics. A similar observation held true between the ensembles *E*(*x* % *N*_p_) and *E*(*x* % *N*_p_, pru). Thus, even strong network pruning did not influence much the quantitative classification metrics, which is good news as it corroborates that ensemble learning with DCNNs of reduced complexity, hence less learnable parameters, was advantageous as it did not influence classification metrics much. Rather, ensemble learning could be performed with much less computational effort and time. A similar conclusion can be drawn with respect to the number of labeled training observations, which could be reduced by a factor of 100 without affecting much the quantitative classification metrics.

**Fig. 5:**
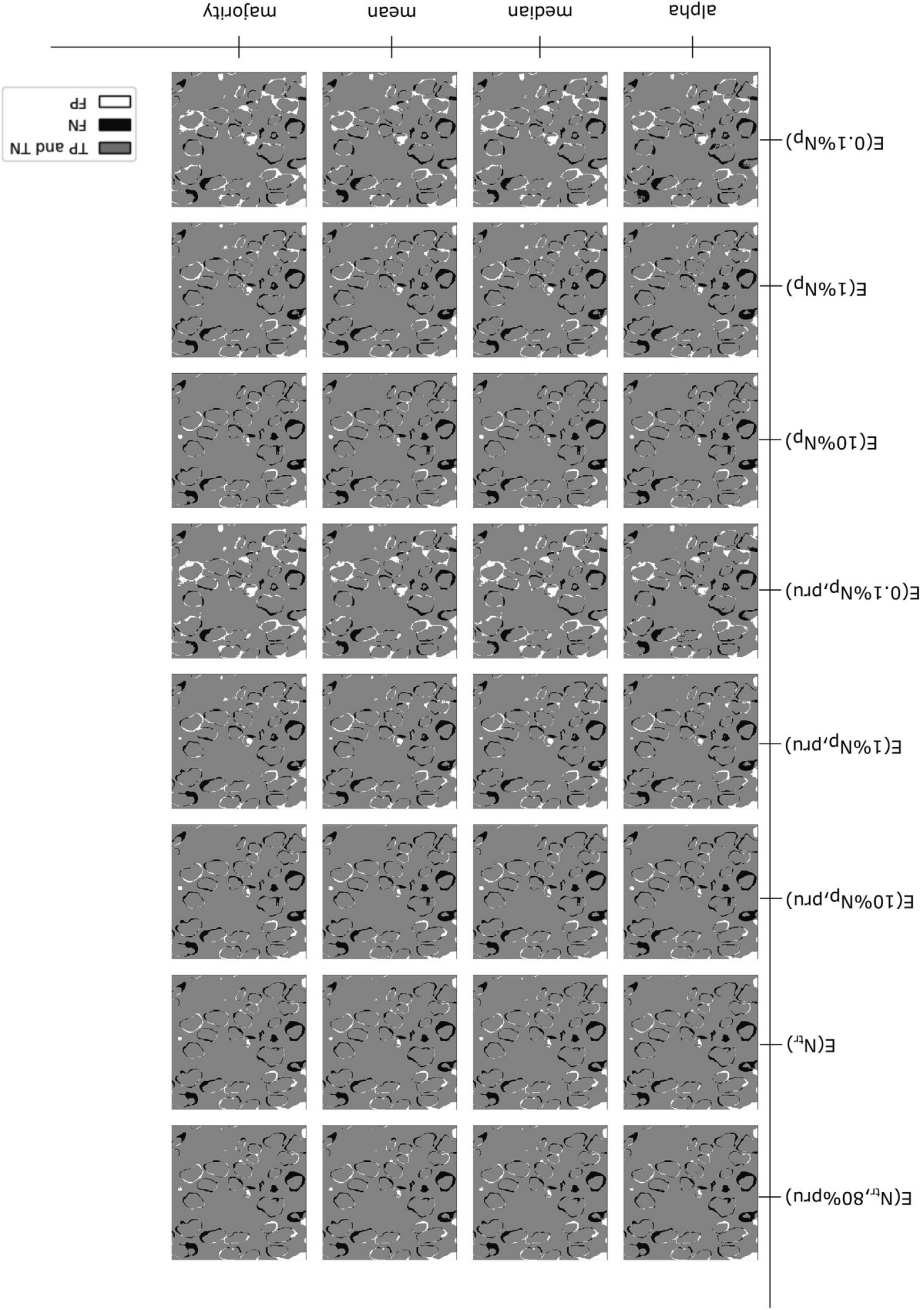
Image patches illustrating the differences between ground truth and prediction CMPs for different ensemble learning and aggregation methods. Gray shades indicate correctly predicted (TP, TN) pixels, black indicates FNs, and white indicates FPs. As expected, especially the border pixels between the foreground objects and the background tend to be classified primarily as FPs or FNs.

In order to render differences between ensemble learning and averaging methods more visible, difference (residual) images between the (manually annotated) ground truth and the predicted segmentation were calculated. Fig. 5 shows difference image patches for one of the test images. The gray color indicates all correctly predicted pixels (TP, TN), the black indicates the FN, and the white indicates FP. As expected, especially the border pixels between the foreground objects and the background tend to yield a lot of false (FP, FN) results. The number of white pixels decreases with the number of used training samples.

Based on the TP, TN, FP, and FN values, the normalized evaluation metrics ACC and DC were then calculated. Both metrics yield values in the range 0 ≤ *ACC*, *DC* ≤ 1. The values are collected in the Table 2 and Table 4. Considering first the training of individual DCNNs while using random seeds for every component network and training them with different training data set sizes, it becomes obvious that the prediction of ACC as well as of DC improve with an increasing number of labeled training observations. Note that ACC also considers TNs, being all background (“*no cell*ˮ) pixels, while DC focuses on the TPs, i.e., the foreground (“*cell*ˮ) pixels. In this specific example data set, the number of background pixels was much larger than the number of foreground pixels, hence the ACC yields values close to *ACC* ≈ 1, while the DC measures the amount of overlap between ground truth and predicted segmentation, hence often resulting in smaller values. Still both measures are close to 1, indicating a successful segmentation of the depicted objects in the micrographs images. Note that although there is some difference in the quantitative statistics ACC vs. cACC and DC vs. cDC, their relative change remains almost the same.

**Table 4:** ACC and DC for different ensemble learning and averaging methods.

Ensemble	Mean value					
	Acc	DC	cAcc	cDC		

*E*(0.1 % *N*_p_)	0.910 ± 0.003	0.648 ± 0.021	0.784 ± 0.033	0.560 ± 0.036		
*E*(1 % *N*_p_)	0.926 ± 0.002	0.781 ± 0.025	0.860 ± 0.020	0.724 ± 0.054		
*E*(10 % *N*_p_)	0.930 ± 0.000	0.792 ± 0.011	**0.877** ± **0.012**	**0.760** ± **0.037**		
*E*(0.1 % *N*_p_, pru)	0.910 ± 0.005	0.652 ± 0.043	0.785 ± 0.035	0.565 ± 0.041		
*E*(1 % *N*_p_, pru)	0.927 ± 0.002	0.785 ± 0.025	0.861 ± 0.020	0.725 ± 0.054		
*E*(10 % *N*_p_, pru)	0.930 ± 0.000	**0.793** ± **0.012**	0.876 ± 0.012	0.759 ± 0.038		
*E*(*N*_tr_)	**0.932** ± **0.003**	0.792 ± 0.024	0.868 ± 0.020	0.742 ± 0.063		
*E*(*N*_tr_, 80 % pru)	**0.932** ± **0.003**	0.792 ± 0.024	0.871 ± 0.021	0.747 ± 0.064

Ensemble	Median				Majority vote	

Acc	DC	cAcc	cDC	Acc	DC

*E*(0.1 % *N*_p_)	0.910	0.660	0.910	0.660	0.910	0.660
*E*(1 % *N*_p_)	0.926	0.783	0.926	0.784	0.926	0.784
*E*(10 % *N*_p_)	0.930	0.793	**0.930**	**0.793**	0.930	0.793
*E*(0.1 % *N*_p_, pru)	0.910	0.664	0.792	0.575	0.910	0.664
*E*(1 % *N*_p_, pru)	0.926	0.785	0.867	0.738	0.926	0.785
*E*(10 % *N*_p_, pru)	0.930	0.793	0.879	0.766	0.930	0.793
*E*(*N*_tr_)	**0.932**	**0.795**	0.877	0.761	**0.932**	**0.795**
*E*(*N*_tr_, 80 % pru)	**0.932**	0.794	0.879	0.765	**0.932**	0.785

Ensemble	Alpha					
	ACC	DC	cACC	cDC		

*E*(0.1 % *N*_p_)	0.909	0.637	0.796	0.575		
*E*(1 % *N*_p_)	0.927	0.777	0.867	0.743		
*E*(10 % *N*_p_)	0.930	**0.790**	**0.880**	**0.770**		
*E*(0.1 % *N*_p_, pru)	0.909	0.641	0.800	0.584		
*E*(1 % *N*_p_, pru)	0.927	0.781	0.868	0.745		
*E*(10 % *N*_p_, pru)	0.930	**0.790**	0.879	0.768		
*E*(*N*_tr_)	**0.932**	0.787	0.874	0.760		
*E*(*N*_tr_, 80 % pru)	**0.932**	0.785	0.876	0.763

Concerning the group of experiments related to a training of the networks of an ensemble *E*(*x* % *N*_p_) with a variable number of training examples and which used random seeds for every individual component network during training, the normalized metrics show similar trends, following those observed with the basic metrics, from whom they were deduced. First, both ACC and DC attain rather high values for all trained ensembles. Also, obviously both metrics improve with increasing number of training data *N*_training_, though to a different extent. The ACC increases by roughly 2% while the DC improves by more than 20%, independent of the ensemble averaging method applied to the individual predictions. Furthermore, both normalized metrics are practically identical in all ensembles across the different ensemble averaging methods, irrespective of the applied network pruning. In any case, the largest fluctuations can be observed for the ensemble *E*(0.1 % *N*_p_) with the least number (*N*_1,training_ = 916) of training examples. Again, the conclusion is that pruning the network complexity did not do any harm to the segmentation performance, hence will be advantageous as it reduces computational complexity for the inference and hence saves computational time.

Last but not least, considering the impact of ensemble learning, the evaluation metrics are higher than those calculated individually, i.e., without an ensemble. For example, if the *N*_1,training_ = 916 training sample was used, the ACC achieved with a single trained DCNN was *ACC* = 0.899 and the DC resulted in *DC*  = 0.674 (see Table 2). In contrast, with an ensemble of 100 networks, trained with the same training sample, the ACC could be increased to *ACC*  =  0.910, but the DC dropped slightly to *DC* = 0.664. Increasing the training data set to *N*_2,training_ = 9 159 training examples and training a single DCNN, the metrics improved to *ACC* = 0.921 and *DC* = 0.768. With an ensemble, however, the metrics further improved to *ACC* = 0.928 and *DC* = 0.785. For the *N*_3,training_ = 91 593 training sample, the metrics further improved from *ACC* = 0.927 and *DC* = 0.788 to *ACC* = 0.930 and *DC* = 0.793. Consequently, the training sets encompassing only *N*_1,training_ = 916 examples did not represent the entire data set adequately, and it was not possible to improve the prediction metrics even when employing ensembles of networks.

### Computational time of ensemble averaging methods

If the computational times for the various ensemble averaging methods are compared with each other, as expected, the alpha-stable fit function is the most complex and the mean value is the fastest. In Table 5, the computation times for the 4 methods are summarized for the inference process. The system used is based on Linux Debian with an Intel i7-7700 CPU (3,6 GHz), a Nvidia GeForce GTX 1070 (8 GB) and 32 GB of RAM.

**Table 5:** Computation time in seconds averaged over 5 predictions and per ensemble averaging method.

Mean	Median	Alpha-stable	Majority vote
2.26 ⋅ 10^−5^	1.33 ⋅ 10^−4^	4.03 ⋅ 10^−2^	1.01 ⋅ 10^−4^

## Conclusion

In this work, different methods were compared to combine individual neural network CMP predictions to a single prediction of an ensemble of trained DCNNs. As combination methods, mean, median, and location parameter of an alpha-stable distribution, fit to the histogram of CMPs, as well as majority voting of the members of an ensemble were used. The study clearly demonstrated that for this specific application example the choice of ensemble averaging method provided only a marginal influence on the applied evaluation metrics (ACC, cACC, DC, and cDC) used to evaluate the segmentation performance. Though representing only a marginal winner for the used example data, a fit of the resulting CMP predictions with an alpha-stable distribution worked best, but with the grain of salt of having the highest computational costs for the inference. Nevertheless, in many practical applications, a simple and fast estimate of the mean of the distribution seems the most proper ensemble averaging method to be used.

In summary, both normalized metrics yielded comparable values across different ensemble learning and averaging methods. Though annoying at first sight, this was an interesting fact as it provided information that reducing network complexity and computational load by network pruning does not harm segmentation performance.

## Funding

This research did not receive any specific grant from funding agencies in the public, commercial, or not-for-profit sectors.

## Conflict of interest

None

## Figures and Tables

**Table 6:** Description of variables used in this publication.

Variable	Description
*N*	Number of models in an ensemble
*N*_*i*_	*i*’s model of an ensemble
*N*_training_	Number of training data
*N*_*i*,training_	Training data sets used to train ensembles
*N*_4,training_	Training data set consists of different numbers of images to train the 100 models of an ensemble. The exact number of training patches is described in Table 8
*N*_3,training_	Training data set of 91 593 images which is a subset of the training data presented in Table 7
*N*_2,training_	Training data set of 9159 images which is a subset of *N*_3,training_
*N*_1,training_	Training data set of 916 images which is a subset of *N*_2,training_
*N*_*p*_	Trainable parameter of 1 single model of 915 934
*E*(0.1 % *N*_p_)	Ensemble with 100 random initialized models trained on *N*_1,training_
*E*(1 % *N*_p_)	Ensemble with 100 random initialized models trained on *N*_2,training_
*E*(10 % *N*_p_)	Ensemble with 100 random initialized models trained on *N*_3,training_
*E*(0.1 % *N*_p_, pru)	Ensemble with 100 pretrained models, each model is pruned by a different amount with the *L*_1_-Norm and finetuned on *N*_1,training_
*E*(1 % *N*_p_, pru)	Ensemble with 100 pretrained models, each model is pruned by a different amount with the *L*_1_-Norm and finetuned on *N*_2,training_
*E*(10 % *N*_p_, pru)	Ensemble with 100 pretrained models, each model is pruned by a different amount with the *L*_1_-Norm and finetuned on *N*_3,training_
*E*(*N*_tr_)	Ensemble with 100 pretrained models and finetuned on *N*_4, training_
*E*(*N*_tr_, 80 % pru)	Ensemble with 100 pretrained models, each model is pruned by 80% with the *L*_1_-Norm and finetuned on *N*_4, training_
*p*	Probability of cell prediction
*p*_*i*_	The i’s probability of cell prediction of the i’s model of an ensemble
*mean*(*p*_*i*_)	Combination of all probabilities of an ensemble with the mean as combination method
*median*(*p*_*i*_)	Combination of all probabilities of an ensemble with the median as combination method
*majority*(*p*_*i*_)	Combination of all probabilities of an ensemble with the majority as combination method
*ϕ*(*ω*)	Characteristic function of *α*-stable distributions
*a*_*i*_	Elements of ground truth to calculate cACC and cDC
*b*_*i*_	Combinations of predictions to calculate cACC and cDC

**Table 7:** A list of images used for pre-training, training, and testing using the data set of Kumar et al.^10^

Patient ID *TCGA*−	Organ	Disease type	Clinic
*Pretraining*			
*A*7 − *A*13*E* − 01*Z* − 00 − *DX*1	Breast	Breast invasive carcinoma	Cristiana Healthcare
*A*7 − *A*13*F* − 01*Z* − 00 − *DX*1	Breast	Breast invasive carcinoma	Cristiana Healthcare

*Training*			
*AR* − *A*1*AK* − 01*Z* − 00 − *DX*1	Breast	Breast invasive carcinoma	Mayo Clinic
*HE* − 7128 − 01*Z* − 00 − *DX*1	Kidney	Kidney renal papillary cell carcinoma	Ontario Institute for Cancer Reasearch
38 − 6178 − 01*Z* − 00 − *DX*1	Liver	Lung adenocarcinoma	University of North Carolina
*G*9 − 6336 − 01*Z* − 00 − *DX*1	Prostate	Prostate adenocarcinoma	Roswell Park
*DK* − *A*2*I*6 − 01*A* − 01 − *TS*1	Bladder	Bladder urothelial carcinoma	Memorial Sloan Kettering
*AY* − *A*8*YK* − 01*A* − 01 − *TS*1	Colon	Colon adenocarcinoma	University of North Carolina
*KB* − *A*93*J* − 01*A* − 01 − *TS*1	Stomach	Stomach adenocarcinoma	University Health Network, Toronto

*Testing*			
*E*2 − *A*1*B*5 − 01*Z* − 00 − *DX*1	Breast	Breast invasive carcinoma	Roswell Park
*B*0 − 5711 − 01*Z* − 00 − *DX*1	Kidney	Kidney renal clear cell carcinoma	University of Pittsburgh
21 − 5784 − 01*Z* − 00 − *DX*1	Liver	Lung squamous cell carcinoma	Fox Chase Cancer Center
*CH* − 5767 − 01*Z* − 00 − *DX*1	Prostate	Prostate adenocarcinoma	Indivumed
*G*2 − *A*2*EK* − 01*A* − 02 − *TSB*	Bladder	Bladder urothelial carcinoma	MD Anderson
*NH* − *A*8*F*7 − 01*A* − 01 − *TS*1	Colon	Colon adenocarcinoma	Candler
*RD* − *A*8*N*9 − 01*A* − 01 − *TS*1	Stomach	Stomach adenocarcinoma	Peter MacCallum Cancer Center

**Table 8:** Two of the ensembles were built by training with a different amount of *N*_training_ = *N*_4,training_. The sizes of training data sets for the 100 neural networks are summarized in this table.

Number	*N*_training_	Number	*N*_training_	Number	*N*_training_	Number	*N*_training_
1	1000	26	17 000	51	65 000	76	190 000
2	1500	27	18 000	52	70 000	77	195 000
3	2000	28	19 000	53	75 000	78	200 000
4	2500	29	20 000	54	80 000	79	210 000
5	3000	30	21 000	55	85 000	80	220 000
6	3500	31	22 000	56	90 000	81	230 000
7	4000	32	23 000	57	95 000	82	240 000
8	4500	33	24 000	58	100 000	83	250 000
9	5000	34	25 000	59	105 000	84	260 000
10	5500	35	27 000	60	110 000	85	270 000
11	6000	36	29 000	61	115 000	86	280 000
12	6500	37	31 000	62	120 000	87	290 000
13	7000	38	33 000	63	125 000	88	300 000
14	7500	39	35 000	64	130 000	89	310 000
15	8000	40	37 000	65	135 000	90	320 000
16	8500	41	39 000	66	140 000	91	330 000
17	9000	42	41 000	67	145 000	92	340 000
18	9500	43	43 000	68	150 000	93	350 000
19	10 000	44	45 000	69	155 000	94	360 000
20	11 000	45	47 000	70	160 000	95	370 000
21	12 000	46	49 000	71	165 000	96	380 000
22	13 000	47	51 000	72	170 000	97	390 000
23	14 000	48	53 000	73	175 000	98	400 000
24	15 000	49	55 000	74	180 000	99	410 000
25	16 000	50	60 000	75	185 000	100	420 000
